# Clinical parameters affecting the therapeutic efficacy of empagliflozin in patients with type 2 diabetes

**DOI:** 10.1371/journal.pone.0220667

**Published:** 2019-08-01

**Authors:** Yun Kyung Cho, Jiwoo Lee, Yu Mi Kang, Jee Hee Yoo, Joong-Yeol Park, Chang Hee Jung, Woo Je Lee

**Affiliations:** 1 Department of Internal Medicine, Asan Medical Center, University of Ulsan College of Medicine, Seoul, Republic of Korea; 2 Department of Internal Medicine, Kangwon National University School of Medicine, Chuncheon, Republic of Korea; 3 International Healthcare Center, Asan Medical Center, University of Ulsan College of Medicine, Seoul, Republic of Korea; 4 Department of Internal Medicine, Samsung Medical Center, Sungkyunkwan University School of Medicine, Seoul, Republic of Korea; International University of Health and Welfare, School of Medicine, JAPAN

## Abstract

We aimed to investigate the clinical factors affecting the therapeutic effectiveness of the sodium–glucose cotransporter-2 inhibitor empagliflozin in patients with type 2 diabetes mellitus (T2DM). We reviewed the medical records of 374 T2DM patients aged between 20 and 75 years who were prescribed empagliflozin 10 mg or 25 mg as add-on therapy for more than 90 consecutive days. Changes in hemoglobin A1c (HbA1c) from baseline levels and the reduction in body weights of the study participants were assessed. We found that younger patients (≤ 50 years), patients with the highest levels of HbA1c (>9%) at baseline, patients with an estimated glomerular filtration rate (eGFR) of >90 mL/min/1.73 m^2^, and patients with a shorter duration of T2DM (< 10 years) were more likely to exhibit a better glycemic response. Multivariate linear regression analysis revealed that a shorter duration of T2DM, higher baseline levels of HbA1c, and higher eGFR were positively associated with HbA1c reduction. Higher BMI and lower HbA1c levels were predictors of a more significant reduction in body weight among patients taking empagliflozin. The glucose-lowering effect of empagliflozin was more evident in T2DM patients with higher baseline HbA1c levels, better renal function, and shorter duration of T2DM.

## Introduction

The prevalence of type 2 diabetes mellitus (T2DM) has steadily increased in Korea in conjunction with the increase in obesity, which has been linked to the adoption of a more Westernized lifestyle [1,2]. Although it is crucial for T2DM patients to exercise adequate glycemic control to prevent hyperglycemia-related complications, many fail to reach their recommended glycemic targets, with only half of the Korean T2DM patients maintaining HbA1c levels of <7.0% [1,3].

Sodium–glucose cotransporter-2 (SGLT2) inhibitors represent a novel class of anti-diabetic medication that aims to improve glucose control by elevating renal glucose excretion by suppressing SGLT2 function in the kidneys [4,5]. SGLT2 inhibitors decrease the fasting plasma glucose (FPG) and postprandial plasma glucose levels, in addition to reducing body weight via secondary caloric loss due to urinary glucose excretion [6,7]. Empagliflozin is an orally active and potent inhibitor of SGLT2. Previous randomized controlled trials reported that empagliflozin is effective as a monotherapeutic medication or in combination with other oral antidiabetic drugs [8–12].

However, only few studies have investigated the factors predictive for an individual’s response to empagliflozin. Previous randomized controlled trials suggested that poor glycemic control at baseline was related to good response to SGLT2 inhibitors [11,13], while renal dysfunction was associated with reduced efficacy of SGLT2 inhibitors in improving glycemic control [14]. Recently, a retrospective study on Korean patients reported that higher baseline HbA1c level and preserved eGFR were predictive markers of better glycemic responses to an another class of SGLT2 inhibitor (dapagliflozin) [15]; specifically, female and obese T2DM patients tended to lose more weight during dapagliflozin treatment. We thus hypothesized that the efficacy of empagliflozin would be also dependent on various clinical characteristics of patients. Although previous studies suggested some parameters that were related to the efficacy of SGLT2 inhibitors, data from real-world practice focusing on the predictors for the efficacy of empagliflozin is still lacking. Therefore, in this retrospective observational study, we aimed to investigate the clinical predictors for the efficacy of empagliflozin.

## Materials and methods

### Ethics statement

In accordance with the ethical guidelines of the Declaration of Helsinki and Korea Good Clinical Practice, this study was approved by the institutional review board of the Asan Medical Center (AMC). The institutional review board waived the requirement to obtain the informed consent from participants, as anonymous archival data without identifying information was used.

### Study population

The data of all patients treated for T2DM at the outpatient clinic of the Department of Internal Medicine at Asan Medical Center (AMC) between November 2016 and December 2017 were reviewed. Those who had been prescribed with empagliflozin (10 mg or 25 mg) for 90 consecutive days or more were included. The median duration of treatment before a follow-up evaluation was 102 days [interquartile range: 91–125 days]. Patients who aged 75 years or more or those who had significant renal impairment [i.e. an estimated glomerular filtration rate (eGFR) < 45mL/min/1.73m^2^] were excluded from the study. Finally, 374 T2DM patients were included as study participants and were assessed for changes in HbA1c level and body weight.

### Clinical and laboratory measurements

Patient data, including age, sex, and time since diabetes diagnosis, were collected by reviewing their electronic medical records. Height and body weight were measured with the participants wearing light clothing and no shoes. The patients’ body mass index (BMI) was calculated by obtaining their body weight in kilograms and dividing it by the square of their height in meters. Blood pressure was measured using an automatic manometer, with the cuff placed on the right arm of the patient, and was obtained after resting for 5 minutes. Blood samples were drawn from the antecubital vein into the vacuum tubes and subsequently analyzed at the certified central laboratory at AMC.

Laboratory serum measurements included hemoglobin A1c (HbA1c), FPG, postprandial 2-hour plasma glucose (PP2), fasting C-peptide, fasting insulin, blood urea nitrogen, creatinine, several lipid parameters, and liver enzymes. Ion-exchange high-performance liquid chromatography (Bio-Rad Laboratories, Inc., Hercules, CA, USA) was used to measure the patients’ HbA1c levels. FPG and PP2 were measured using the immunoturbidimetric method and the enzymatic colorimetric method on a Toshiba 200 FR auto-analyzer (Toshiba Medical Systems Co., Ltd., Tokyo, Japan), respectively. Serum C-peptide and insulin concentrations were obtained by performing immunoradiometric assays (TFB, Tokyo, Japan). Fasting total cholesterol, high-density lipoprotein cholesterol (HDL-C), low-density lipoprotein cholesterol (LDL-C), triglycerides (TGs), aspartate aminotransferase (AST), and alanine aminotransferase (ALT) were measured by enzymatic colorimetry using a Toshiba 200 FR Neo analyzer (Toshiba Medical Systems Co., Ltd., Tokyo, Japan). All enzyme activities were measured at 37°C. The eGFR was determined using the CKD-EPI equation.

### Statistics

Continuous variables were expressed as the mean ± standard deviation (SD). Categorical variables were expressed as percentages. Student’s t-tests and one-way analysis of variance with Scheffe’s method were used to assess the continuous variables, while chi-squared tests were used to assess the categorical variables. Linear regression analysis was performed to assess the relationship between the clinical parameters and empagliflozin efficacy. Pearson’s correlation coefficients were used to evaluate the association between changes in HbA1c and body weight. All statistical analyses were performed using SPSS software (version 20.0 for Windows; SPSS, Inc., Chicago, IL). *P* <0.05 was used to represent statistical significance for all analyses described above.

## Results

### Baseline characteristics of the study participants

The characteristics of the patients enrolled in this study are presented in Table 1. Their mean age was 54.2 ± 9.8 years, and 61.7% of the patients were men. The mean baseline HbA1c level and BMI were 8.4 ± 1.4% and 28.5 ± 4.2 kg/m^2^, respectively.

**Table 1:** Baseline characteristics of the study participants.

Variables	Total (n = 374)
**Age, years**	54.2 ± 9.8
**Male**	232 (61.7)
**T2DM duration, years**	9.4 ± 7.4
**SBP, mmHg**	133.7 ± 16.8
**DBP, mmHg**	76.0 ± 10.9
**Body mass index, kg/m**^**2**^	28.5 ± 4.2
**HbA**_**1c**_**, %**	8.4 ± 1.4
**FPG, mg/dL**	169.9 ± 60.0
PP2, mg/dL*	231.9 ± 82.7
Fasting C-peptide, ng/mL*	2.7 ± 2.0
Fasting insulin, μU/mL*	17.3 ± 30.7
HOMA-IR*	6.8 ± 10.4
HOMA-B*	84.6 ± 196.5
**Total cholesterol, mg/dL**	154.6 ± 40.3
**Triglycerides, mg/dL**	178.6 ± 134.7
**HDL cholesterol, mg/dL**	46.4 ± 10.5
**LDL cholesterol, mg/dL**	97.7 ± 30.5
**BUN, mg/dL**	15.5 ± 5.3
**Creatinine, mg/dL**	0.83 ± 0.21
**eGFR, mL/min/1.73 m**^**2**^	93.1 ± 16.6
**AST, IU/L**	29.6 ± 18.4
**ALT, IU/L**	33.2 ± 23.9
Urine ACR, mg/g Cr*	137.0 ± 570.1

Data are presented as mean ± standard deviation or as n (%)

*PP2 levels were not available for 140 patients. Fasting C-peptide levels were not available for 74 patients. Fasting insulin, HOMA-IR and HOMA-B levels were not available for 197 patients. Urine ACR levels were not available for 78 patients.

T2DM: type 2 diabetes mellitus, SBP, systolic blood pressure, DBP, diastolic blood pressure, FPG: fasting plasma glucose, PP2: postprandial 2-h glucose, HOMA-IR/-B: homoeostasis model assessment for insulin resistance/beta-cell function, HDL/LDL: high-density/low-density lipoprotein, BUN: blood urea nitrogen, eGFR: estimated glomerular filtration rate, AST/ALT, aspartate/alanine aminotransferase, ACR: albumin/creatinine ratio

### Clinical parameters affecting the glucose-lowering effect of empagliflozin

**Fig 1:**
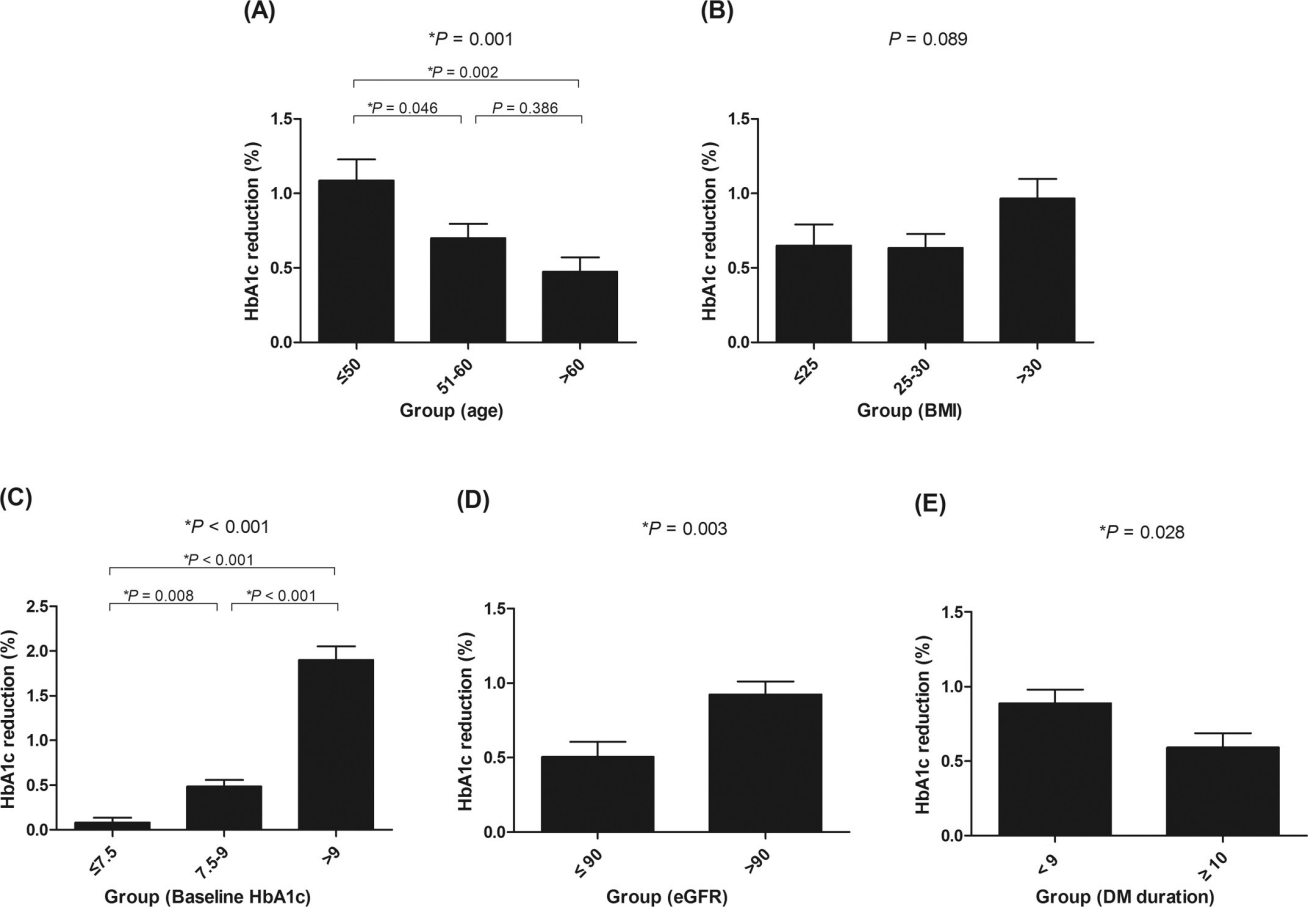
Subgroup analyses of the changes in glycated hemoglobin (HbA1c) levels according to (A) age, (B) body mass index, (C) initial HbA1c, (D) estimated glomerular filtration, and (E) diabetes mellitus duration.

To elucidate the clinical factors affecting the glucose-reducing effect of empagliflozin, subgroup analyses were performed (Fig 1). When the participants were grouped by age, the decrease in HbA1c levels tended to be more substantial in younger participants than in older participants. HbA1c levels fell by 1.1% in patients aged ≤50 years (n = 118), 0.7% in patients aged 51–60 years (n = 153), and 0.5% in patients aged >60 years (n = 103; Fig 1A), with the difference between the youngest and oldest groups being statistically significant (*P* = 0.001). For the subgroups with BMI ≤25 kg/m^2^ (n = 71), 25–30 kg/m^2^ (n = 185), and >30 kg/m^2^ (n = 100), HbA1c levels decreased by 0.6%, 0.6%, and 1.0%, respectively (Fig 1B); these changes were not significantly different (*P* = 0.089). Finally, patients with higher baseline HbA1c levels exhibited a significantly greater reduction in HbA1c during follow-up examinations (Fig 1C). In patients with baseline HbA1c levels of <7.5% (n = 119), HbA1c fell by only 0.1%. By contrast, reductions in HbA1c of 0.5% and 1.9% were observed in patients with baseline HbA1c levels of 7.5–9.0% (n = 149) and > 9% (n = 106), respectively. These intergroup differences were all statistically significant. In a subsequent analysis based on eGFR, HbA1c decreased by 0.5% in patients with an eGFR of ≤90 mL/min/1.73 m^2^ (n = 135) and by 0.9% in patients with an eGFR of >90 mL/min/1.73 m^2^ (n = 232; Fig 1D), and the difference was statistically significant (*P* = 0.003). When analyzed by time since T2DM diagnosis, patients with a shorter duration of T2DM (<10 years, n = 213) had a significantly greater fall in HbA1c levels (0.9%) than those with a longer duration of T2DM (0.6%, ≥10 years, n = 159, *P* = 0.028; Fig 1E).

Multivariate regression analyses were also conducted to identify the markers that can predict the effects of empagliflozin on HbA1c levels (Table 2). Single regression analysis found that the reduction in HbA1c levels was associated with younger age, shorter T2DM duration, higher baseline HbA1c levels, and higher eGFR levels. Shorter T2DM duration, higher baseline HbA1c levels, and higher eGFR levels were also identified as significant independent predictors of greater decrease in HbA1c levels in patients taking empagliflozin after multiple regression analysis with adjustments for age, T2DM duration, baseline HbA1c levels, and eGFR levels (Table 2).

**Table 2:** Multiple regression analysis of the changes in HbA1c levels in patients treated with empagliflozin.

*HbA1c change*	Unadjusted B (P)	Adjusted* B (P)
**Age, years**	0.029 (0.007)	-0.001 (0.827)
**Gender, female**	0.149 (0.277)	
**Body mass index, kg/m**^**2**^	-0.026 (0.110)	
**T2DM duration, years**	0.029 (0.001)	0.023 (0.004)
**HbA**_**1c**_**, %**	-0.578 (<0.001)	-0.584 (<0.001)
**eGFR**	-0.016 (<0.001)	-0.012 (<0.001)

*Adjusted for age, T2DM duration, baseline HbA1c, and eGFR

eGFR: estimated glomerular filtration rate, HbA1c: hemoglobin A1c, T2DM, type 2 diabetes mellitus

We have additionally analyzed the effectiveness of empagliflozin 10 mg (n = 175) or 25 mg separately (n = 199). Higher baseline HbA1c level was a significant parameter for a better glycemic response in both of empagliflozin 10 mg and 25 mg group (S1 Fig and S1 Table). A shorter duration of T2DM and preserved renal function were significantly associated with more HbA1c reduction in empagliflozin 25 mg group (S1 Fig and S1 Table).

In a subsequent analysis of HbA1c reduction in patients taking empagliflozin, those who exhibited a reduction in HbA1c levels (≥10%) from baseline were classified as “responders.” When the baseline characteristics of responders and non-responders were compared, responders demonstrated significantly shorter T2DM histories, poorer glycemic control before empagliflozin treatment, and higher eGFR levels. Homeostatic model assessment for insulin resistance (HOMA-IR) scores, total cholesterol, and LDL cholesterol levels were also higher among responders than among non-responders (S2 Table).

### Clinical parameters associated with body weight reduction in patients taking empagliflozin

**Fig 2:**
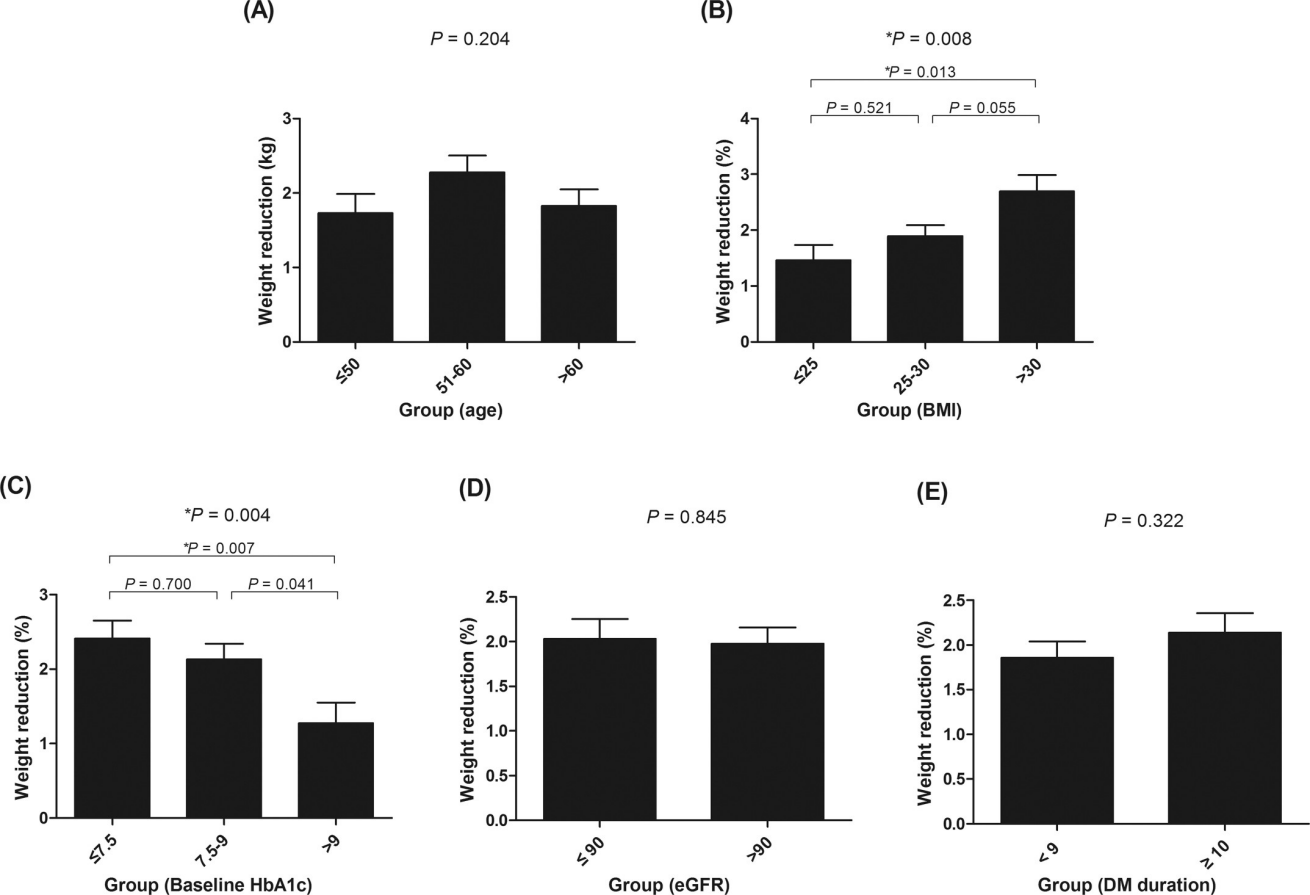
Subgroup analyses of the changes in body weight (kg) according to (A) age, (B) body mass index, (C) initial hemoglobin A1c, (D) estimated glomerular filtration, and (E) diabetes mellitus duration.

We assessed the clinical parameters associated with the reduction in body weight in patients taking empagliflozin. To evaluate the clinical factors affecting changes in body weight, subgroup analyses were performed (Fig 2). When the participants were grouped by age, no significant difference in the loss of body weight was observed (*P* = 0.204; Fig 2A). When the participants were grouped by their baseline BMI, patients with a BMI ≤25 kg/m^2^ (n = 71) lost 1.5 kg, those with a BMI 25–30 kg/m^2^ (n = 185) lost 1.9 kg, and those with a BMI >30 kg/m^2^ (n = 100; Fig 2B) lost 2.7 kg. Obese participants with a BMI >30 kg/m^2^ had a significant decrease in body weight compared with those with a BMI ≤25 kg/m^2^ (*P* = 0.013). Moreover, body weight reduction was significantly associated with baseline HbA1c levels (*P* = 0.004; Fig 2C). Body weight fell by an average of 2.4 kg in patients with baseline HbA1c levels of <7.5% (n = 119) and 2.1 kg in those with baseline HbA1c levels of 7.5%–9.0% (n = 149). In contrast, body weight decreased by only 1.3 kg in patients with baseline HbA1c levels of >9% (n = 106). Intergroup differences were significant between the group with HbA1c levels of <7.5% and that with HbA1c levels of >9% (*P* = 0.017) and between the group with HbA1c levels of 7.5%–9.0% and that with HbA1c levels of >9% (*P* = 0.041; Fig 2C). Finally, no significant differences were observed among the eGFR level or T2DM duration subgroups (*P* = 0.845 and *P* = 0.322; Fig 2D and 2E, respectively).

Multivariate regression analyses were also conducted to identify predictive markers for the reduction in body weight in T2DM patients taking empagliflozin (Table 3). The reduction in body weight was associated with a higher baseline BMI and lower baseline HbA1c levels in the unadjusted analysis. In the multiple regression analysis adjusted for BMI and baseline HbA1c levels, both BMI and baseline HbA1c levels remained significant independent predictors of a better response to empagliflozin (*P* = 0.010 and *P* < 0.001, respectively).

**Table 3:** Multiple regression analyses of the changes in body weight in patients treated with empagliflozin.

*Body weight reduction*	Unadjusted B (P)	Adjusted* B (P)
**Age, years**	-0.004 (0.014)	
**Gender, female**	-0.329 (0.250)	
**Body mass index, kg/m**^**2**^	-0.086 (0.009)	-0.084 (0.010)
**T2DM duration, years**	-0.019 (0.320)	
**HbA**_**1c**_**, %**	0.400 (<0.001)	0.387 (<0.001)
**eGFR**	-0.001 (0.899)	

*Adjusted for BMI and baseline HbA1c.

eGFR: estimated glomerular filtration rate, HbA1c: hemoglobin A1c, T2DM, type 2 diabetes mellitus

In our additional analyses according to the dose of empagliflozin, patients with a higher BMI at baseline lose more weight than lean patients in both of empagliflozin 10 mg and 25 mg group (S2 Fig and S3 Table). Lower baseline HbA1c was significantly related to more body weight reduction by empagliflozin 10 mg (S2 Fig and S3 Table).

In a follow-up analysis, T2DM patients who experienced a reduction in body weight (≥3% of their baseline weight) after taking empagliflozin were classified as responders. A comparison of the baseline characteristics of the responders and non-responders found that responders had lower baseline HbA1c levels than non-responders. HOMA-IR scores were also higher in non-responders than in responders (S4 Table).

### Correlation between HbA1c and body weight reduction

A correlation analysis was conducted to assess the relationship between changes in HbA1c levels and body weight among T2DM patients taking empagliflozin. No significant association was found between the two outcomes (r = -0.018, *P* = 0.742; S3 Fig).

### Safety and tolerability

Adverse events (AEs) included hypoglycaemia, genital infection, urinary tract infection, urinary frequency or nocturia, ketoacidosis, and hypersensitivity reaction. The overall incidence of treatment-related AEs is shown in S5 Table. In total, 10.4% of participants experienced AEs after empagliflozin treatment. AEs leading to treatment discontinuation were reported for 7 patients, of which 5 cases were due to genital infection and 2 cases were due to urinary tract infection. Totally, 15 patients (4.0%) experienced genital infection and 2 patients (0.5%) experienced urinary tract infection. Sixteen patients (4.3%) reported hypoglycaemia; however, there was no severe hypoglycemic event requiring assistance or medical intervention. There were no reports of hypersensitivity reaction, acute pancreatitis, diabetic ketoacidosis, or death.

## Discussion

In the present study, we found that higher baseline HbA1c levels, better renal function, and shorter T2DM duration were predictive markers for more effective lowering of glucose levels using empagliflozin. In addition, the predictive clinical parameters for body weight reduction were higher BMI and lower baseline HbA1c levels.

Previous studies have reported the clinical predictors of glycemic response to SGLT2 inhibitors [11,13,16,17]. The amount of glucose excreted by SGLT2 inhibitors increases as the plasma glucose concentration increases, which explains why the glucose-lowering effect of an SGLT2 inhibitor was more powerful in patients with poor glycemic control [17,18]. In a phase-III trial comparing empagliflozin monotherapy with sitagliptin in T2DM patients, greater decrease in HbA1c levels were observed in empagliflozin-treated patients with the highest baseline HbA1c levels [11]. In a randomized controlled trial that evaluated the effects of a combination of empagliflozin and linagliptin in T2DM patients, higher baseline HbA1c levels were predictive of the higher clinical efficacy of empagliflozin [13]. In a recent study in Korea, T2DM patients with poor glucose control were more likely to benefit from treatment with the SGLT2 inhibitor dapagliflozin [16]. Another retrospective study in Japan also reported that high baseline HbA1c levels were an independent predictor of the glucose-lowering effect of SGLT2-inhibitor therapy [17]. Consistent with previous studies, our findings showed that the reduction in HbA1c levels in patients taking empagliflozin was associated with high baseline HbA1c levels.

The glucose-lowering effect of SGLT2 inhibitors is due to their glucosuric effect in the kidneys, where SGLT2 is extracellularly inhibited in the lumen of the proximal tubule when the SGLT2 inhibitors are filtered by the glomerulus [19]. Compared with placebo, SGLT2 inhibitors did not improve the glycemic control in patients with moderate renal impairment [20]. Therefore, it can be speculated that SGLT2 inhibitors are only effective in individuals with healthy renal function. In fact, the Korean and Japanese studies mentioned above both found that good renal function was another independent predictive marker for better glycemic response to SGLT2 inhibitors [16,17]. Our findings add further evidence that good renal function can act as a predictive marker for better responses to empagliflozin therapy.

Moreover, our findings–which indicated that shorter T2DM duration is a predictor of better glycemic responses to empagliflozin–is in agreement with those of previous studies. An ASSIGN-K study found that SGLT2 inhibitors were more effective in patients with a shorter duration of diabetes and in those with high baseline HbA1c levels [21]. As explained in ASSIGN-K study, one possible explanation for this is that the efficacy of SGLT2 inhibitors may be reduced in patients with a longer disease duration due to the upregulation of SGLT2 expression or the elevation of the renal glucose threshold [21]. Further studies are needed to identify the predictive role and underlying mechanisms of disease duration on the effect of SGLT2 inhibitors in T2DM patients.

The prevalence of diabetes mellitus is increasing worldwide, reaching epidemic proportions due to the rising levels of obesity [22]. The mean BMI of Korean diabetic patients is gradually increasing, and recent studies have found that half of the patients in Korea with diabetes are obese [1,2]. In this vein, SGLT2 inhibitors are a promising therapeutic option as the use of these inhibitors can result in weight loss in addition to better glycemic control [23]. We found that empagliflozin was associated with weight loss in T2DM patients, with an average body weight reduction of 2.0 ± 2.7 kg from baseline levels. We also found that patients with a higher baseline BMI lost more weight within the observation period (Fig 2B), although baseline BMI was not associated with better glycemic control (Fig 1B). In addition, there was no significant correlation between decrease in HbA1c and decrease in body weight among patients taking empagliflozin (S3 Fig). These findings were in agreement with those of previous studies, which used the SGLT2 inhibitor dapagliflozin [16]. Baseline BMI was not significantly associated with glycemic improvement by dapagliflozin. Additionally, no significant correlation was observed between dapagliflozin’s glucose-lowering effects and loss of body weight. This study suggested that compensatory hyperphagia in obese patients could be a possible explanation for this observation [16]. The overexpression or overactivation of SGLT2 in obese T2DM patients could be another possible explanation for the variations in the effectiveness of SGLT2 inhibitors [16].

Another important finding from our study is that higher baseline HbA1c levels were associated with less weight reduction (Fig 2C). One possible explanation for this is that insulin resistance may affect the patient’s response to empagliflozin therapy. Interestingly, while the difference was not statistically significant, HOMA-IR levels tended to be higher in the HbA1c responder group and lower in the body weight responder group (S1 and S2 Tables). Therefore, it is possible that severe insulin resistance is related to better glycemic control and a reduction in the rate of weight loss in patients taking empagliflozin. However, as the HOMA-IR data of several study participants were unavailable, further studies are needed to elucidate the role of HOMA-IR on the effectiveness of SGLT2 inhibitors.

This study has several limitations. First, this was a retrospective study without a placebo group. Second, only Korean patients were included in this study. Third, patients’ lifestyles including diet and exercise were not monitored. Fourth, the content of the meal to measure postprandial plasma glucose levels has not been standardized. Lastly, the follow-up period was relatively short; therefore, we could not assess the long-term effects of empagliflozin treatment. Despite these limitations, we have revealed indicators of glycaemic responses and weight loss with empagliflozin treatment. Our finding could suggest some guidance on prescribing empagliflozin to T2DM patients for clinicians. Furthermore, this retrospective observational study used data based on practice in a real-world clinical setting, which is meaningful in a clinical aspect.

## Conclusion

In summary, the present study revealed that the glucose-lowering effect of empagliflozin was more evident in T2DM patients with higher baseline HbA1c levels, better renal function, and shorter T2DM duration. In addition, a higher BMI and lower baseline HbA1c levels were predictive clinical parameters of body weight reduction in patients treated with empagliflozin.

## Supporting information

S1 Fig Subgroup analyses of the changes in glycated hemoglobin (HbA1c) levels according to age, body mass index, initial HbA1c, estimated glomerular filtration, and diabetes mellitus duration in empagliflozin (EMPA) 10mg users (A-E) and EMPA 25mg users (F-J).
(TIF)

S2 Fig Subgroup analyses for changes in body weight (kg) according to age, body mass index, initial HbA1c, estimated glomerular filtration, and diabetes mellitus duration in empagliflozin (EMPA) 10mg users (A-E) and EMPA 25mg users (F-J).
(TIF)

S3 Fig Correlation analysis of changes in HbA1c and body weight. (TIF)

S1 Table Multiple regression analysis for changes in HbA1c levels in empagliflozin (EMPA) 10mg users (A) and EMPA 25mg users (B).
(DOCX)

S2 Table Baseline characteristics of the study participants according to empagliflozin response. **A responder was defined as a patient with an HbA1c reduction ≥ 10% of baseline HbA1c levels.** Data are presented as mean ± standard deviation or as n (%).
(DOCX)

S3 Table Multiple regression analyses for changes in body weight in patients in empagliflozin (EMPA) 10mg users (A) and EMPA 25mg users (B).
(DOCX)

S4 Table Baseline characteristics of the study participants according to empagliflozin response. **A responder was defined as those patients exhibiting a body weight reduction of ≥ 3% their baseline body weight.** Data are presented as mean ± standard deviation or as n (%).
(DOCX)

S5 Table Summary of adverse events during study period. (DOCX)
