# Supplementary figures and images for: Clinical parameters affecting the therapeutic efficacy of empagliflozin in patients with type 2 diabetes
Source: PLoS One. 2019 Aug 1;14(8):e0220667. doi: 10.1371/journal.pone.0220667 (PMC6675078; doi:10.1371/journal.pone.0220667)

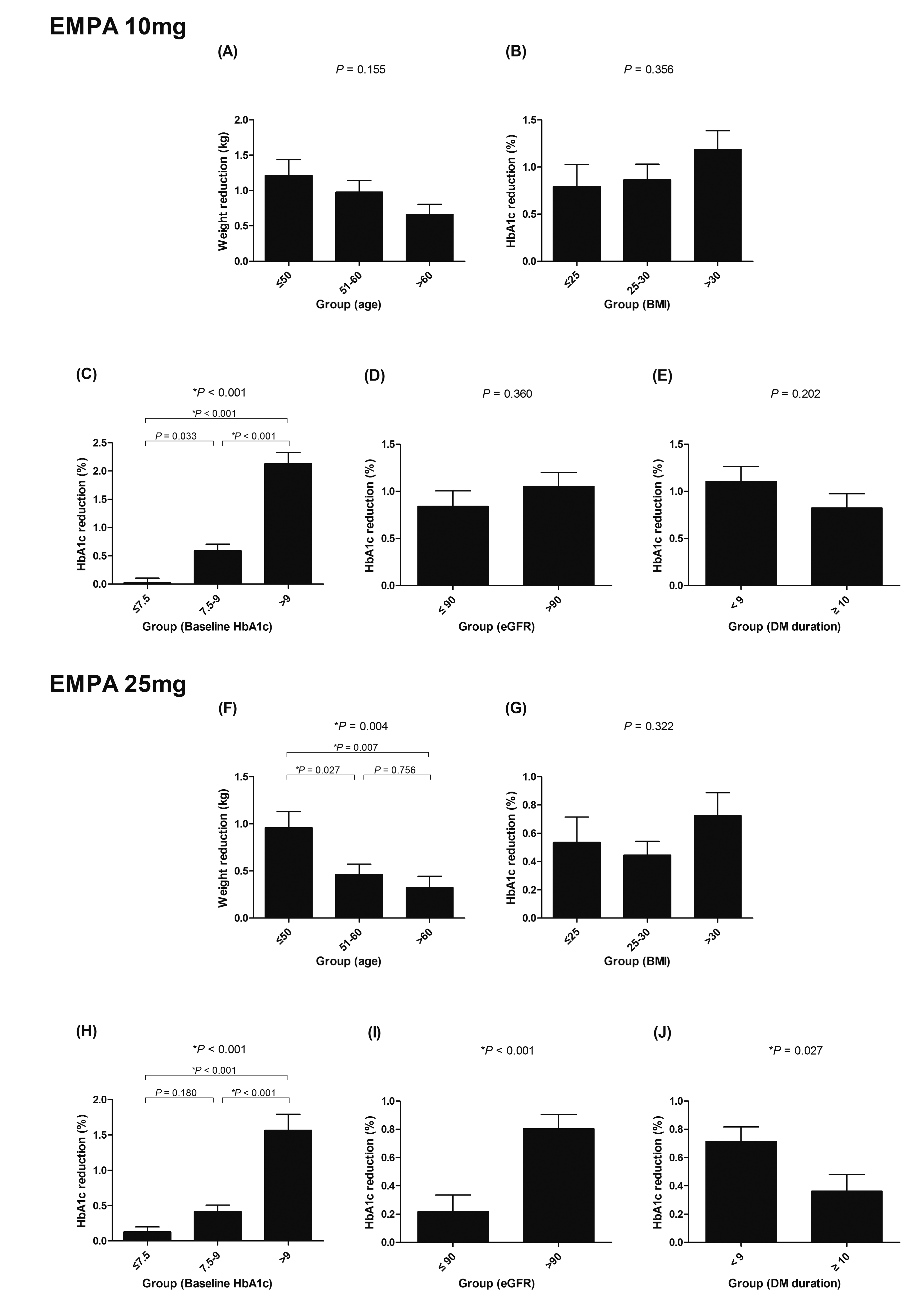

Supplement: S1 Fig — Subgroup analyses of the changes in glycated hemoglobin (HbA1c) levels according to age, body mass index, initial HbA1c, estimated glomerular filtration, and diabetes mellitus duration in empagliflozin (EMPA) 10mg users (A-E) and EMPA 25mg users (F-J). (TIF) [file pone.0220667.s001.tif]

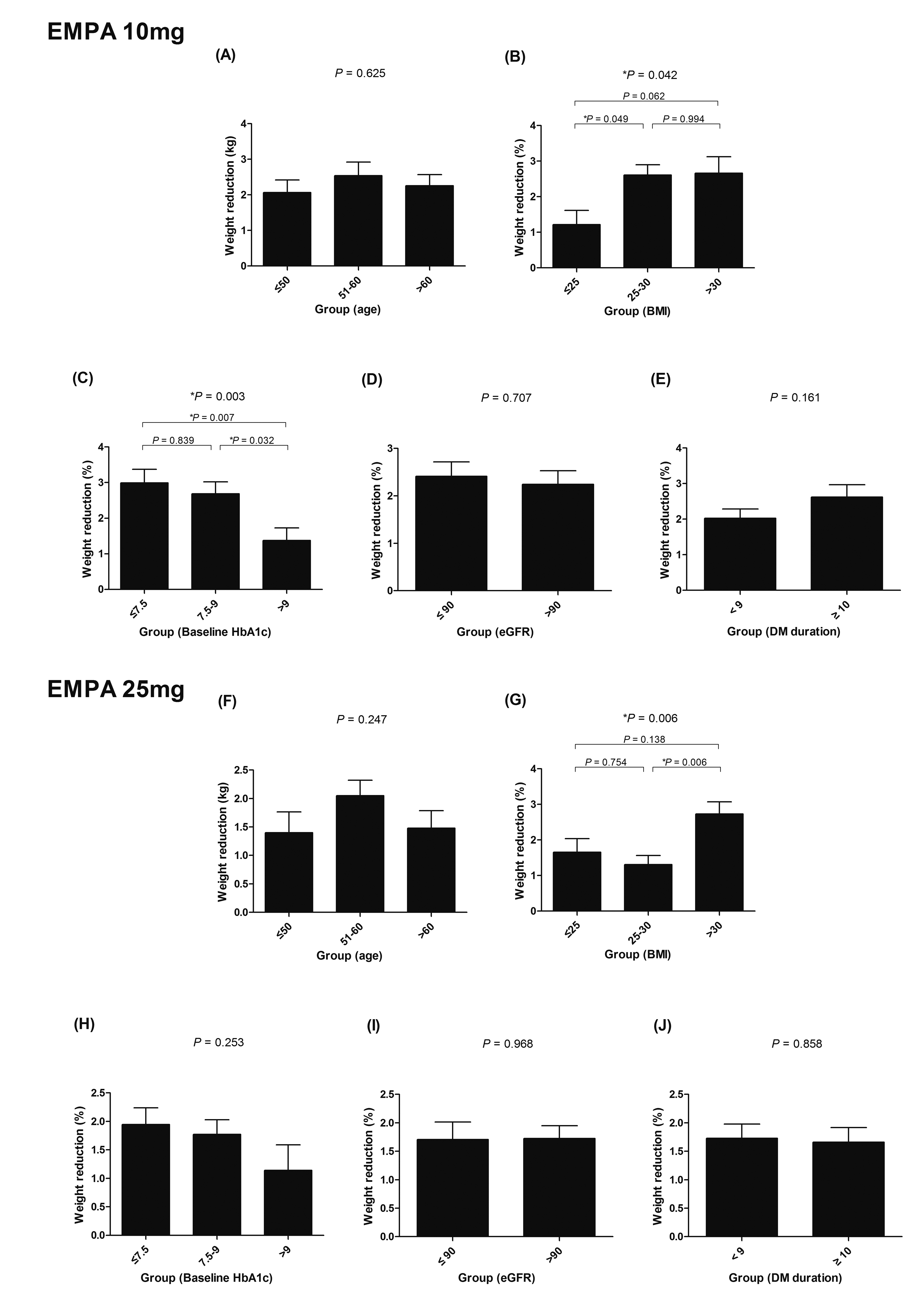

Supplement: S2 Fig — Subgroup analyses for changes in body weight (kg) according to age, body mass index, initial HbA1c, estimated glomerular filtration, and diabetes mellitus duration in empagliflozin (EMPA) 10mg users (A-E) and EMPA 25mg users (F-J). (TIF) [file pone.0220667.s002.tif]

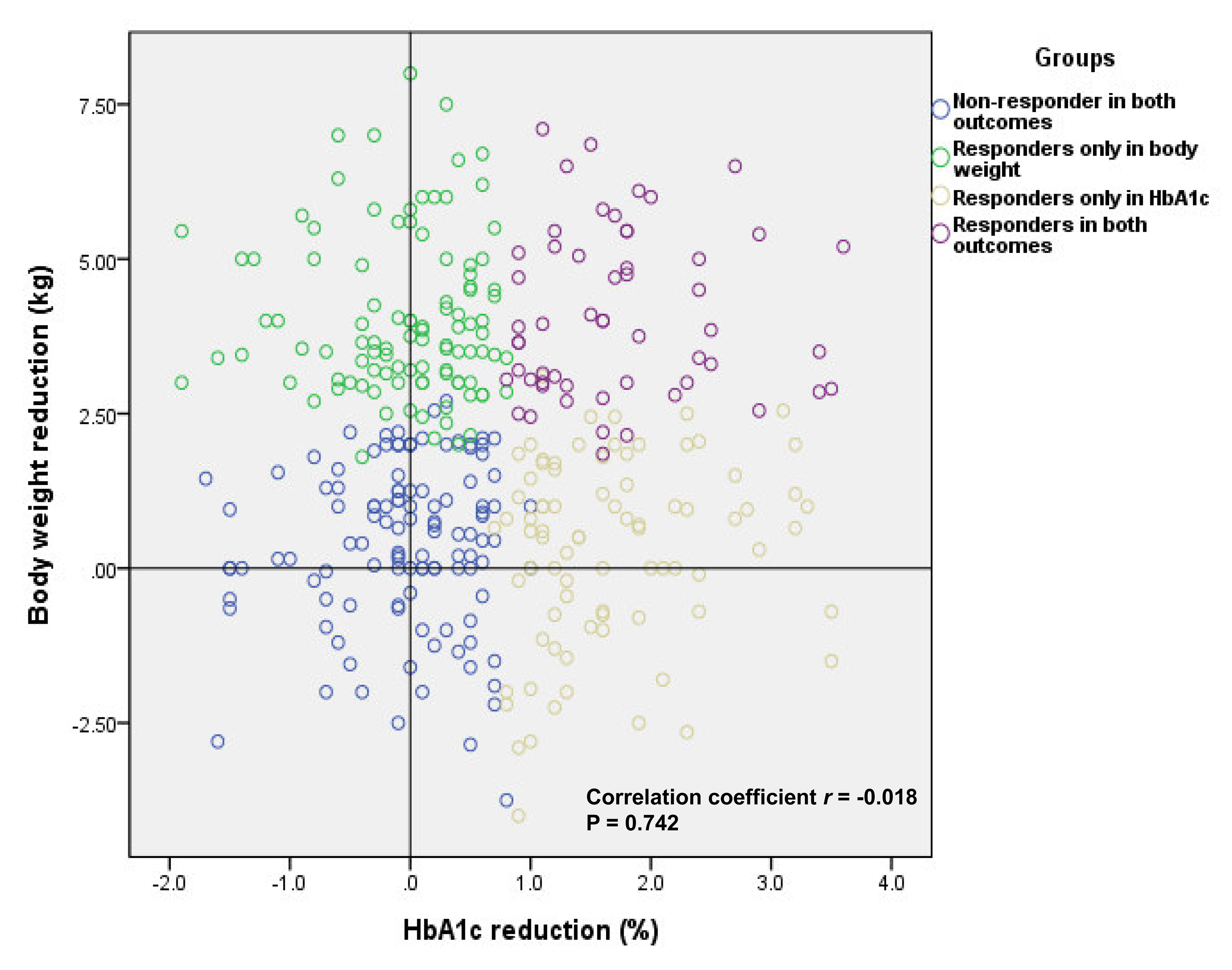

Supplement: S3 Fig — (TIF) [file pone.0220667.s003.tif]
