# Supplementary material for: Clinical parameters affecting the therapeutic efficacy of empagliflozin in patients with type 2 diabetes
Source: PLoS One. 2019 Aug 1;14(8):e0220667. doi: 10.1371/journal.pone.0220667 (PMC6675078; doi:10.1371/journal.pone.0220667)
Supplement: S1 Table — Multiple regression analysis for changes in HbA1c levels in empagliflozin (EMPA) 10mg users (A) and EMPA 25mg users (B). (DOCX) [file pone.0220667.s004.docx]

**S1 Table. Multiple regression analysis for changes in HbA1c levels in empagliflozin (EMPA) 10mg user (A) and EMPA 25mg user (B).**

**(A) EMPA 10mg**

| ***HbA1c change*** | **Unadjusted**  **B (P)** | **Adjusted***  **B (P)** |
| --- | --- | --- |
| **Age, years** | 0.026 (0.014) | -0.004 (0.708) |
| **Gender, female** | 0.378 (0.221) |  |
| **Body mass index, kg/m^2^** | -0.032 (0.299) |  |
| **T2DM duration, years** | 0.030 (0.032) | 0.027 (0.028) |
| **HbA_1c_, %** | -0.613 (<0.001) | -0.620 (<0.001) |
| **eGFR** | -0.011 (0.086) | -0.008 (0.164) |

*Adjusted for age, T2DM duration, baseline HbA1c, and eGFR

**(B) EMPA 25mg**

| ***HbA1c change*** | **Unadjusted**  **B (P)** | **Adjusted***  **B (P)** |
| --- | --- | --- |
| **Age, years** | 0.030 (0.008) | -0.001 (0.987) |
| **Gender, female** | -0.025 (0.879) |  |
| **Body mass index, kg/m^2^** | -0.021 (0.207) |  |
| **T2DM duration, years** | 0.031 (0.005) | 0.020 (0.045) |
| **HbA_1c_, %** | -0.496 (<0.001) | -0.495 (<0.001) |
| **eGFR** | -0.022 (<0.001) | -0.018 (<0.001) |

*Adjusted for age, T2DM duration, baseline HbA1c, and eGFR
