# Supplementary material for: Clinical parameters affecting the therapeutic efficacy of empagliflozin in patients with type 2 diabetes
Source: PLoS One. 2019 Aug 1;14(8):e0220667. doi: 10.1371/journal.pone.0220667 (PMC6675078; doi:10.1371/journal.pone.0220667)
Supplement: S2 Table — A responder was defined as a patient with an HbA1c reduction ≥ 10% of baseline HbA1c levels. Data are presented as mean ± standard deviation or as n (%). (DOCX) [file pone.0220667.s005.docx]

**S2 Table. Baseline characteristics of the study participants according to empagliflozin response. A responder was defined as a patient with an HbA1c reduction ≥ 10% of baseline HbA1c levels.**

| **Variables** | **Responder  (n = 152)** | **Non-responder  (n = 222)** | **P** |
| --- | --- | --- | --- |
| **Age, years** | 53.1 ± 10.4 | 55.0 ± 9.3 | 0.070 |
| **Male** | 93 (61.2) | 138 (62.2) | 0.914 |
| **T2DM duration, years** | 8.5 ± 7.3 | 10.1 ± 7.5 | 0.041 |
| **SBP, mmHg** | 134.3 ± 17.0 | 133.2 ± 16.6 | 0.561 |
| **DBP, mmHg** | 76.5 ± 11.6 | 75.7 ± 10.4 | 0.717 |
| **Body mass index, kg/m^2^** | 29.0 ± 4.7 | 28.1 ± 3.8 | 0.064 |
| **HbA_1c_, %** | 9.2 ± 1.3 | 7.8 ± 1.1 | <0.001 |
| **FPG, mg/dL** | 185.8 ± 64.9 | 159.1 ± 53.8 | <0.001 |
| **PP2, mg/dL*** | 247.2 ± 81.7 | 223.6 ± 82.3 | 0.037 |
| **Fasting C-peptide, ng/mL*** | 2.9 ± 1.8 | 2.6 ± 2.1 | 0.256 |
| **Fasting insulin,**$\boldsymbol{\mu}$**U/mL*** | 20.1 ± 38.7 | 15.5 ± 24.3 | 0.382 |
| **HOMA-IR*** | 8.8 ± 14.0 | 5.6 ± 7.0 | 0.042 |
| **HOMA-B*** | 81.0 ± 187.4 | 86.9 ± 202.9 | 0.844 |
| **Total cholesterol, mg/dL** | 162.0 ± 45.9 | 149.6 ± 35.1 | 0.003 |
| **Triglycerides, mg/dL** | 194.0 ± 177.0 | 168.6 ± 97.1 | 0.119 |
| **HDL cholesterol, mg/dL** | 45.5 ± 10.4 | 47.0 ± 10.6 | 0.173 |
| **LDL cholesterol, mg/dL** | 102.8 ± 34.8 | 94.5 ± 27.0 | 0.012 |
| **BUN, mg/dL** | 15.2 ± 5.4 | 15.7 ± 5.3 | 0.380 |
| **Creatinine, mg/dL** | 0.80 ± 0.19 | 0.86 ± 0.22 | 0.010 |
| **eGFR, mL/min/1.73 m^2^** | 96.5 ± 15.9 | 90.9 ± 16.8 | 0.001 |
| **AST, IU/L** | 31.0 ± 21.5 | 28.7 ± 15.9 | 0.231 |
| **ALT, IU/L** | 35.7 ± 26.9 | 31.4 ± 21.5 | 0.092 |
| **Urine ACR, mg/g Cr*** | 133.0 ± 375.7 | 139.6 ± 665.8 | 0.913 |

Data are presented as mean ± standard deviation or as n (%)

*PP2 levels were not available for 140 patients. Fasting C-peptide levels were not available for 74 patients. Fasting insulin, HOMA-IR and HOMA-B levels were not available for 197 patients. Urine ACR levels were not available for 78 patients.

T2DM: type 2 diabetes mellitus, SBP, systolic blood pressure, DBP, diastolic blood pressure, FPG: fasting plasma glucose, PP2: postprandial 2-h glucose, HOMA-IR/-B: homoeostasis model assessment for insulin resistance/beta-cell function, HDL/LDL: high-density/low-density lipoprotein, BUN: blood urea nitrogen, eGFR: estimated glomerular filtration rate, AST/ALT, aspartate/alanine aminotransferase, ACR: albumin/creatinine ratio
