# Supplementary material for: Clinical parameters affecting the therapeutic efficacy of empagliflozin in patients with type 2 diabetes
Source: PLoS One. 2019 Aug 1;14(8):e0220667. doi: 10.1371/journal.pone.0220667 (PMC6675078; doi:10.1371/journal.pone.0220667)
Supplement: S3 Table — Multiple regression analyses for changes in body weight in patients in empagliflozin (EMPA) 10mg users (A) and EMPA 25mg users (B). (DOCX) [file pone.0220667.s006.docx]

**S3 Table. Multiple regression analyses for changes in body weight in patients in empagliflozin (EMPA) 10mg users (A) and EMPA 25mg users (B).**

**(A) EMPA 10mg**

| ***Body weight reduction*** | **Unadjusted**  **B (P)** | **Adjusted***  **B (P)** |
| --- | --- | --- |
| **Age, years** | -0.012 (0.559) |  |
| **Gender, female** | -0.180 (0.678) |  |
| **Body mass index, kg/m^2^** | -0.139 (0.017) | -0.139 (0.013) |
| **T2DM duration, years** | -0.044 (0.110) |  |
| **HbA_1c_, %** | 0.513 (<0.001) | 0.503 (<0.001) |
| **eGFR** | 0.005 (0.667) |  |

*Adjusted for BMI and baseline HbA1c.

**(B) EMPA 25mg**

| ***Body weight reduction*** | **Unadjusted**  **B (P)** | **Adjusted***  **B (P)** |
| --- | --- | --- |
| **Age, years** | -0.003 (0.870) |  |
| **Gender, female** | -0.413 (0.273) |  |
| **Body mass index, kg/m^2^** | -0.056 (0.150) | -0.054 (0.167) |
| **T2DM duration, years** | 0.012 (0.654) |  |
| **HbA_1c_, %** | 0.285 (0.069) | 0.279 (0.078) |
| **eGFR** | -0.008 (0.442) |  |

*Adjusted for BMI and baseline HbA1c.
