# Supplementary material for: Clinical parameters affecting the therapeutic efficacy of empagliflozin in patients with type 2 diabetes
Source: PLoS One. 2019 Aug 1;14(8):e0220667. doi: 10.1371/journal.pone.0220667 (PMC6675078; doi:10.1371/journal.pone.0220667)
Supplement: S4 Table — A responder was defined as those patients exhibiting a body weight reduction of ≥ 3% their baseline body weight. Data are presented as mean ± standard deviation or as n (%). (DOCX) [file pone.0220667.s007.docx]

**S4 Table. Baseline characteristics of the study participants according to empagliflozin response. A responder was defined as those patients exhibiting a body weight reduction of ≥ 3% their baseline body weight.**

| **Variables** | **Responder  (n = 161)** | **Non-responder  (n = 206)** | **P** |
| --- | --- | --- | --- |
| **Age, years** | 54.3 ± 9.8 | 54.1 ± 9.8 | 0.814 |
| **Male** | 93 (56.7) | 138 (65.7) | 0.086 |
| **T2DM duration, years** | 9.5 ± 7.3 | 9.4 ± 7.4 | 0.885 |
| **SBP, mmHg** | 134.1 ± 16.5 | 133.4 ± 17.0 | 0.721 |
| **DBP, mmHg** | 76.1 ± 11.2 | 76.0 ± 10.8 | 0.950 |
| **Body mass index, kg/m^2^** | 28.7 ± 3.8 | 28.3 ± 4.5 | 0.463 |
| **HbA_1c_, %** | 8.2 ± 1.2 | 8.5 ± 1.5 | 0.010 |
| **FPG, mg/dL** | 167.1 ± 56.4 | 172.2 ± 62.7 | 0.412 |
| **PP2, mg/dL*** | 237.7 ± 85.8 | 227.3 ± 80.2 | 0.342 |
| **Fasting C-peptide, ng/mL*** | 2.6 ± 1.4 | 2.8 ± 2.3 | 0.231 |
| **Fasting insulin,**$\boldsymbol{\mu}$**U/mL*** | 13.3 ± 14.8 | 20.4 ± 38.5 | 0.094 |
| **HOMA-IR*** | 5.2 ± 5.1 | 8.1 ± 13.0 | 0.045 |
| **HOMA-B*** | 67.5 ± 139.8 | 97.7 ± 237.8 | 0.313 |
| **Total cholesterol, mg/dL** | 154.2 ± 41.0 | 155.0 ± 39.8 | 0.841 |
| **Triglycerides, mg/dL** | 170.6 ± 127.3 | 184.9 ± 140.2 | 0.316 |
| **HDL cholesterol, mg/dL** | 46.8 ± 10.9 | 46.1 ± 10.3 | 0.545 |
| **LDL cholesterol, mg/dL** | 98.5 ± 31.7 | 97.2 ± 29.6 | 0.680 |
| **BUN, mg/dL** | 15.4 ± 5.6 | 15.5 ± 5.1 | 0.887 |
| **Creatinine, mg/dL** | 0.82 ± 0.2 | 0.84 ± 0.2 | 0.278 |
| **eGFR, mL/min/1.73 m^2^** | 93.7 ± 15.6 | 92.8 ± 17.4 | 0.619 |
| **AST, IU/L** | 31.4 ± 20.6 | 28.3 ± 16.5 | 0.105 |
| **ALT, IU/L** | 34.2 ± 24.6 | 32.4 ± 23.3 | 0.467 |
| **Urine ACR, mg/gCr*** | 74.5 ± 259.9 | 189.5 ± 732.5 | 0.065 |

Data are presented as mean ± standard deviation or as n (%)

*PP2 levels were not available for 140 patients. Fasting C-peptide levels were not available for 74 patients. Fasting insulin, HOMA-IR and HOMA-B levels were not available for 197 patients. Urine ACR levels were not available for 78 patients.

T2DM: type 2 diabetes mellitus, SBP, systolic blood pressure, DBP, diastolic blood pressure, FPG: fasting plasma glucose, PP2: postprandial 2-h glucose, HOMA-IR/-B: homoeostasis model assessment for insulin resistance/beta-cell function, HDL/LDL: high-density/low-density lipoprotein, BUN: blood urea nitrogen, eGFR: estimated glomerular filtration rate, AST/ALT, aspartate/alanine aminotransferase, ACR: albumin/creatinine ratio
