# Supplementary material for: Clinical parameters affecting the therapeutic efficacy of empagliflozin in patients with type 2 diabetes
Source: PLoS One. 2019 Aug 1;14(8):e0220667. doi: 10.1371/journal.pone.0220667 (PMC6675078; doi:10.1371/journal.pone.0220667)
Supplement: S5 Table — (DOCX) [file pone.0220667.s008.docx]

**S5 Table. Summary of adverse events during study period**

|  | **Empagliflozin (n = 374)** |
| --- | --- |
| Total AEs | 39 (10.4) |
| AEs leading to discontinuation | 7 (1.8) |
| **Special interest categories** |  |
| Hypoglycemia | 16 (4.3) |
| Genital infection | 15 (4.0) |
| Urinary tract infection | 2 (0.5) |
| Urinary frequency or nocturia | 5 (1.3) |
| Ketoacidosis | 0 |
| Hypersensitivity reaction | 0 |
| Deaths | 0 |

Data are expressed as number (%).
